# Unraveling the regional environmental ecology dominated baijiu fermentation microbial community succession and associated unique flavor

**DOI:** 10.3389/fmicb.2024.1487359

**Published:** 2024-10-31

**Authors:** Xiaowei Wu, Xiaoli Zhao, Li Wang, Bi Chen, Fangzhou Li, Zhi Tang, Fengchang Wu

**Affiliations:** ^1^Jiangsu Key Laboratory of Atmospheric Environment Monitoring and Pollution Control, School of Environmental Science and Engineering, Nanjing University of Information Science and Technology, Nanjing, China; ^2^State Key Laboratory of Environmental Criteria and Risk Assessment, Chinese Research Academy of Environmental Sciences, Beijing, China; ^3^Kweichow Moutai Distillery Co., Ltd., Renhuai, China; ^4^Chishui River Middle Basin, Watershed Ecosystem, Observation and Research Station of Guizhou Province, Guiyang, China

**Keywords:** food microbiology, Jiang-flavor-Baijiu, fermentation, microbial community, environmental ecology

## Abstract

Chinese baijiu as one of the famous distilled liquor in which fermented in open environments, with various microorganisms (i.e., bacteria, fungi, and yeast) involved in their brewing process, and created corresponding unique flavor. However, the sources of environmentally enriched microbial communities associated with liquor fermentation are still being characterized yet. Given the dependence of microbial growth and reproduction on environmental ecology, it is important to understand the correlation between baijiu fermentation microbial community and surrounding environmental ecology (i.e., temperature, humidity, wind, and precipitation). This study systematically overviewed the sources of microorganisms in the Jiang-flavor-Baijiu fermentation system. The results showed that microorganisms in baijiu brewing (i.e., mold, lactic acid bacteria, and yeast) mainly originated from surrounding environmental matrices, including the air (i.e., *Yeast*, *Streptomyces* and *Bacillus*), soil (i.e., *Xanthomonas*, *Methanococcus* and *Comamonas*) and water (i.e., *Flavobacterium*, *Acinetobacter*, and *Pseudomonas*) via atmospheric transport, raw material transfer and surface runoff. In addition, the unique baijiu fermentation microbial community diversity depends on local geology and meteorological conditions, highlighting that the structural stability and diversity of the microorganisms in the Baijiu brewing process dominated by local environmental ecology. We also explored the regional environmental conditions on the microbial community and found that the unique Jiang-flavor-Baijiu fermentation microbial community diversity depends on local geology and meteorological conditions. The Jiang-flavor-Baijiu workshop is located in the basin of the middle-and low latitude mountainous areas, with sufficient solar irradiation and rainfall, high air humidity, and low wind speed that favor the growth and propagation of Baijiu fermentation microorganisms. Therefore, the obtained conclusions provide new insights unraveling the key factor controlling the unique flavor of Chinese Baijiu, where protecting the ecology of baijiu brewing-regions is fundamental for maintaining the long-term quality of baijiu.

## 1 Introduction

Chinese Jiang-flavor Baijiu, a kind of fermented beverage (approximately 40–60% alcohol by volume) obtained by solid-state fermentation distillation with a mixture of various microorganisms and enzymes as starter, which popular around the world owing to its unique flavor (Zhou et al., 2024; Zhu et al., 2024). Jiang-flavor-Baijiu is produced by the fermentation of raw grains (sorghum and wheat) to carboxylic acids, alcohols, aldehydes, and esters through several stages: Daqu-making, heap fermentation (on the ground), and pit fermentation (in sealed mud pits) (Liu and Sun, 2018; Tu et al., 2022). Studies revealed that highly complex microbial community of fermenters are important determinants of Baijiu flavor and quality (Dai et al., 2019; Zhang et al., 2022), as the flavor components (e.g., alcohols, aldehydes, carboxylic acids and esters) are microbial (e.g., yeast, mold, lactic acid bacteria, and *Bacillus*) fermentation products (Tu et al., 2022; Xu et al., 2021; Xu et al., 2022). *Zygosaccharomyces* produce 2-ethylhexanol, decanoic acid, dodecanol, lauric acid, octanoic acid, 2-nonanol, acetophenone, ethyl caprylate, 4-tert-butylphenol and other complex trace flavor components during baijiu brewing (Zhuang et al., 2017). *Pichia anomala* produces acetic acid, octanoic acid, lactic acid, phenylethyl alcohol, 2,3-butanediol, ethyl myristate, and 2-furanethanol during fermentation, all of which contributes to the flavor of Baijiu (Kong et al., 2014). These Baijiu fermentation associated microorganisms predominate several fermentation processes, such as Daqu (Wang P. et al., 2017; Zheng et al., 2014), raw materials (Du et al., 2019; Wang Q. et al., 2021), and fermented grains (Ao et al., 2020; Beecroft et al., 2012). However, the sources of Baijiu fermentation microorganisms remain unclear, particularly regarding their origins within the fermentation facilities (fermentation conditions and specific raw materials) (Pang et al., 2018; Wang et al., 2022b; Zheng et al., 2014). Given the adaptability of different microorganisms to environmental conditions such as temperature, wind, precipitation, and humidity, it is important to understand how highly variations in the environment influence the structure and stability of Baijiu fermentation microbial populations (Dai et al., 2019; Du et al., 2019; Zhu et al., 2015). Maintaining a stable microbial fermentation community structure is closely related to the stability of Baijiu flavor; therefore, it is important to understand the correlation between the environmental ecology of Baijiu-making region and fermentation associated raw materials and processing (Li et al., 2016).

Environmental factors that impact the Jiang-flavor-Baijiu fermentation microbial communities include temperature, humidity, wind, precipitation, geography, and sunlight irradiation, which located on the Renhuai, Guizhou province China (106°22′ E, 27°51′ N) (Knights et al., 2011). Jiang-flavor-Baijiu fermentation workshops are surrounding by abundant mountains and are considered as a typical basin area. The unique basin environment crates a relative slow air velocity in the Baijiu manufacturing region (wind speed: 1.3–1.35 m/s, the maximum and minimum wind speed is only 0.05 m/s), thereby stabilizing the Baijiu fermentation microbial community structure. The stability of the microbial community structure in the Baijiu fermentation area aids in maintaining the stability of Baijiu quality. The region in which Jiang-flavor-Baijiu manufactured has a subtropical climate with warm winter and hot summers (annual average temperature is 17.7°C), year-round humidity (annual precipitation is 800 ~ 1,000 mm), and plenty of sunshine irradiation (annual duration is 1,400 h), which provides a suitable habitat for the growth and reproduction of fermentation microbes (Wu et al., 2014). The Jiang-flavor-Baijiu region also has a unique geological structure and climatic conditions, with unique regional soil, water, and air quality that directly influence the dominant microbial community (Li et al., 2017). The mechanism by which these environmental factors influence the Jiang-flavor Baijiu fermentation microbial community structure has not been clarified.

When interpreting the Baijiu “flavor code,” the following issues need to be considered: (i) clarifying the ecological characteristics necessary for a high-quality Baijiu manufacturing workshop (i.e., temperature, climate, humidity, precipitation, and topography); (ii) establishing the correlation between the ecological characteristics of Baijiu fermentation and microbial community structure; and (iii) exploring the mechanisms by which Baijiu fermentation microbiota from the surrounding environment (soil, air, or water) enter the fermentation process. This review systematically summarizes the sources of environmentally enriched microbial communities and the ecological conditions influencing the Jiang-flavor-Baijiu fermentation microbial communities during solid state fermentation. The findings described here will provide insights into the intricate relationship between the environmental ecology and the composition (or stability) of the Jiang-flavor-Baijiu fermentation microbial community structure. At the same time, we advocate more efforts to be conducted to protect the ecological environment quality of baijiu-making areas, maintain the stability of baijiu-making microbial community structure, so as to ensure and enhance the flavor of liquor.

## 2 Characterizing the influence of microbial diversity on Paijiu quality and flavor

**Figure 1:**
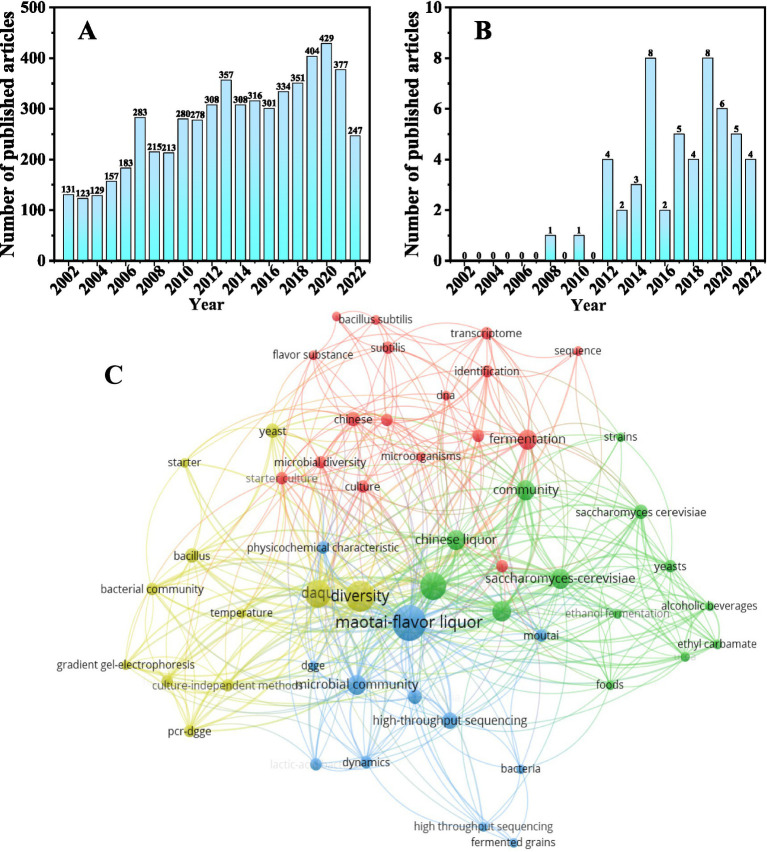
(A) Statistics of the number of published literatures on liquor microbiota from 2002 to 2022; (B) 2002–2022 Jiang-flavored baijiu microbial impact study published literature over the years statistics; (C) Research Progress of Microorganism in Jiang-flavored baijiu Brewing VOSviewer (1.6.16) Analysis (Different radius of the ball in the figure represents the keyword frequency, the greater the radius of the ball, the higher the keyword frequency).

It is important to clarify the relationship between environmental parameters, fermentation microbial community structure, and Baijiu flavor and quality to optimize processes and screen the most efficient fermentation microbiota. We reviewed current knowledge on the impact of fermentation microbiota on Baijiu quality and flavor, by performing a literature search for articles published in the past 20 years (2002–2022) on the literature database Web of Science, with the keywords “Baijiu, microorganisms.” Between January 2002 and March 2023, a total of 4,718 papers were published, 3,732 (65.19%) of which were published in the past 10 years, reflecting a substantial increase in research interest in the microorganisms associated with Baijiu flavor and quality (Figure 1A).

The general research areas into the effects of microbiota on Jiang-flavor-Baijiu quality and flavor were searched in the Web of Science database using “Jiang-flavor Baijiu, microbiota.” Between January 2002 and March 2023, 53 publications addressed the effect of fermentation microbiota on Jiang-flavor-Baijiu, 60.37% of which were published in the past 5 years, reflecting increased interest (Figure 1B). Subsequently, VOS viewer 1.6.16 was used to visualize the prevailing research on the influence of Baijiu fermentation microbiota on flavor. A total of 280 keywords were sorted by the frequency; 53 keywords that appeared at least twice were selected. Prior studies of the microbial effects on Baijiu flavor have focused on the effects of Daqu and solid-state fermentation on microbial community structure, while the environmental factors driving the spatial–temporal variations in microbial community structure during stacking fermentation remain unknown (Figure 1C).

## 3 Microbial communities in Jiang-flavor-baijiu fermentation systems and their impact on baijiu quality

### 3.1 Occurrence of Jiang-flavor-baijiu fermentation of microbes in raw material, high-temperature Daqu, and fermented grains

**Figure 2:**
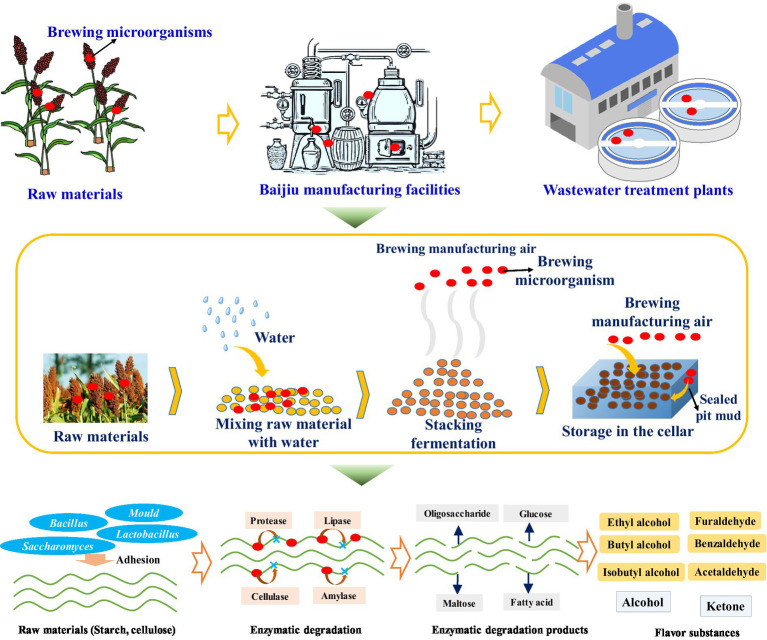
Critical impact of environmental microbiota on the production of Baijiu flavor compounds.

**Figure 3:**
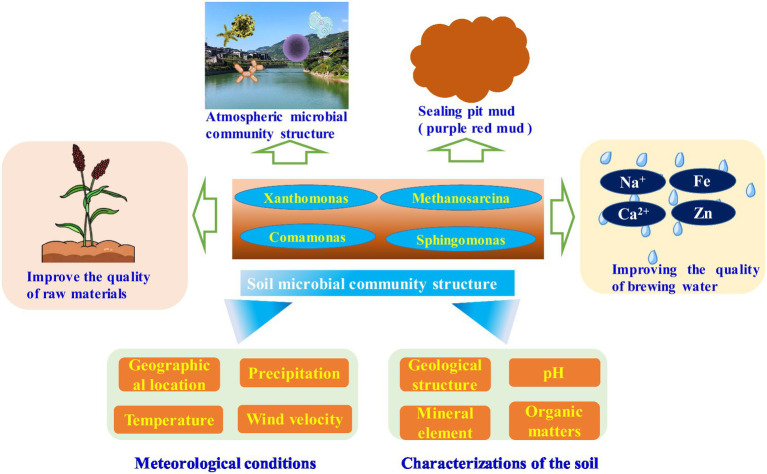
Soil microbial community structure in Jiang-flavor liquor brewing area and its effect on liquor flavor.

During fermentation of Jiang-flavor-Baijiu, several complex microbial communities (i.e., bacteria, yeasts, and molds) are readily involved and mainly residue in the raw material (sorghum), heap fermented grains, Daqu, and pit mud (Figure 2) (Yang et al., 2020a,b). In terms of heap fermentation, microbiota become enriched on the surface of the raw materials and produce Baijiu-flavor precursors such as alcohols, carboxylic acids, and esters (Tang et al., 2021). The core communities in the fermented grain liquor starters include lactic acid bacteria and *Bacillus* (*Bacillus amyloliquefaciens*, *Bacillus licheniformis* and *Lactobacillus* sp.), as well as yeast (*Saccharomyces cerevisiae*, *Candida*, *Hansenula polymorpha*, *Hansenula anomala*, and *Pichia pastoris*), *Aspergillus* (*Aspergillus*, *Monascus*, *Aspergillus flavus*, *Monascus purpureus*, *Aspergillus niger*, *Aspergillus nidulans,* and *Aspergillus fumigatus*), *Thermomyces*, *Rhizopus,* and *Mucor* (*Mucor plentus*, *Mucor pusillus*, *Mucor racemosus*, and *Mucor racemosus*) (Fan et al., 2006) (see Figure 3).

High-temperature Daqu is a typical starter for Jiang-flavor-Baijiu as the saccharification and fermentation starter (Wu et al., 2023). The microbial community structure of Daqu is important for developing quality and flavor as the primary microbial and enzyme source (Zuo et al., 2020). The microbiota such as *Aspergillus, Thermoactinomyces, Lentibacillus, Bacillus, Kroppenstedtia, Staphylococcus, and Saccharopolyspora* in Daqu decompose macromolecular substances in fermented grains, providing aroma substances or precursors of aroma substances that determine the flavor of Baijiu (Wang et al., 2020). Daqu can be categorized as low-temperature Daqu (peak temperature < 50°C), medium-temperature Daqu (peak temperature range in 50–60°C), and high-temperature Daqu (peak temperature > 60°C) (Jin et al., 2020). Given the adaptability of Baijiu fermentation microbiota to temperature, microbial community structure in Baijiu Daqu varies with temperature category. For example, bacteria rather than fungi predominate in Jiang-flavor-Baijiu Daqu, such as *Thermoactinomyces*, *Saccharopolyspora*, *Streptomyces*, *Brevibacterium*, *Staphylococcus*, *Lentibacillus*, *Kroppenstedtia* and *Bacillus* (Wang et al., 2020). These microbes are adapted to high temperatures and represent the unique diversity of Jiang-flavor Baijiu.

In the fermented grains, the fungi *Ascomycota*, *Zygomycota*, *Chytridiomycota,* and *Basidiomycota* are most prevalent; *Ascomycota* accounts for 94% at the phylum level. At the genus level, *Aspergillus*, *Emericella*, *Candida*, *Monascus*, *Pichia*, *Wallemia*, *Trichosporon,* and *Wickerhamomyces* are the primary contributors (Table 1). The predominant fungi in Baijiu fermented grains are *Aspergillus* (2.70–50.02%) and *Monascus* (15.08–52.47%). Bacteria and fungi residue in raw material, Daqu, and fermented grains secrete enzymes (glucoamylase, liquefying enzyme, and protease) that decompose starch, protein, and other macromolecular substances into flavor substances (Wang et al., 2008; Wang M. Y. et al., 2018; Yang et al., 2019). Thus, the microbial community structure in Baijiu fermentation is an important determinant of Baijiu quality and flavor. The correlation between Baijiu fermentation microbial community and their quality is subsequently reviewed.

**Table 1:** Different stages of brewing process (sand making, stacking fermentation and koji making) dominant microbial populations (bacteria, yeast, and fungi).

	Pit fermentation	Fermented grains	Daqu
Bacteria			
*Acinetobacter*	1	4,6,14	15
*Lactobacillus*	1	2,4,5,6,7,8	3,7,10,11,13,16,17
*Weissella*	1	6	13
*Pediococcus*		2,3,6	3
*Lentibacillus*		3,8	12
*Kroppenstedtia*		2,3,4,8,14	3
*Acetobacter*		4,14	3,7
*Bacillus (Bacillus subtilis, Bacillus licheniformis, Clostridium)*	1	4,6,7,8,9	6,7,11,12,13,15,16,7,17
*Oceanobacillus*	1	2,3,4,8	
*Virgibacillus*		2,4,6	
*Sphingomonas*		4	
*Thermoactinomyces*		8	3,12,13
*Desmospora*		3	
*Enterobacter*		3	10
*Halomonas*			3
*Saccharopolyspora*			3,13
*Weissela*		3	3
*Staphylococcus*		2,4,6	6,12
*Mitsuokella*			
*Pediococcus*	1	3,4	3
*Enterococcus*		8	
*Enterobacter*		8	
*Kocuria*		2	
*Aerobacter*		2	
Fungi and yeast			
*Pichia kudriavzevii*	1	3,6,8,9,18	4,12,18,19
*Hansenula anomala*			17
*Hansenula polymorpha*	1		4
*Zygosaccharomyces bailii*		5,8	12,18
*Wickerhamomyces*			13
*Saccharomyces*	1	3,5,7,8,9,18	4,12,14,16,18,19
*Thermoascus*		2,3,6,7,9	13,15
*Aspergillus*	1	2,3,5,6,7,9,10,11	11,13,15,19
*Monascus*		3,7	13,15
*Thermomyces*		2,3,5,7,9	13,15,20
*Candida apicola*		2,18	4,19,20
*Byssochlamys*		7,11	11
*Rhizopus*		6,10,11	7,11,13,15,19
*Mucor*			11,19
*Eurotium*		5	
*Penicillium*		5	
*Torulaspora (Torulopsis)*		7	19
*Schizosaccharomyces*		8	18
*Saccharomyces*		7	
*Monascus*		5,7,11	17,19
*Byssochlamys*		5,7	13,15
*Streptomyces*			16

The microbiota listed in the figure only give information in the existing literature and do not represent all microbial populations; the numbers in the figure represent the corresponding reference numbers, as follows: Bacteria: 1. Liu et al. (2019); 2. Hao et al. (2021); 3. Wang (2022); 4. Zhang et al. (2021); 5. Liu et al. (2022b); 6. Zhuansun et al. (2022); 7. Wang et al. (2008); 8. Wang H. et al. (2021); 9. Wang M. Y. et al. (2018); 10. Jin et al. (2019); 11. Gan et al. (2019); 12. Wang et al. (2020); 13. Zuo et al. (2020); 14. Zhu et al. (2022); 15. Li et al. (2014); 16. Liu et al. (2012); 17. Wang et al. (2015b); Fungi and yeast: 1. Liu et al. (2019); 2. Zhu et al. (2022); 3. Zhang et al. (2021); 4. Wang et al. (2008); 5. Wang M. Y. et al. (2018); 6. Zhuansun et al. (2022); 7. Wang H. et al. (2021); 8. Wu et al. (2013); 9. Hao et al. (2021); 10. Chen et al. (2014); 11. Wang (2022); 12. Wu et al. (2012b); 13. Ding et al. (2024); 14. Gan et al. (2019); 15. Jiang et al. (2021); 16. Wang et al. (2020); 17. Wang et al. (2016); 18. Wu et al. (2013); 19. Wang M. Y. et al. (2018); 20. Jin et al. (2019).

### 3.2 Microbe mediated Jiang-flavor-baijiu fermentation and formation of flavor compounds

Owing the difference in fermentation raw materials and technical procedure, the microbial community structure and metabolism procedure of Jiang-flavor-Baijiu are varied in comparison with other types of Chinese Baijiu (i.e., Laobaigan-flavored baijiu, Fuyu-flavored baijiu, and Feng-flavored baijiu), which vary in their decomposition of starch, cellulose, and polysaccharides from raw materials (Wang X. et al., 2017; Wang X. D. et al., 2017; Wang Z. et al., 2018). For example, *Lactobacillus* promotes the Maillard reaction and produces lactic acid-related organic acids and diacetyl via sugar fermentation (Yang et al., 2020b). *Pichia anomala*, a saccharomycete, generates acetic acid, octanoic acid, lactic acid, phenylethyl alcohol, 2,3-butanediol, ethyl tetradecanoate, and 2-furanethanol (Kong et al., 2014). *Saccharomyces cerevisiae* produces *β*-glucosidase during fermentation process and further promotes the release of monoterpenes from glycoside precursors (Zha et al., 2018). Thus, many flavor substances are detected in Baijiu, which contribute to distinctive scent and flavor profiles (Table 2). Jiang-flavor-Baijiu has a complex characteristic scent generated by esters (ethyl acetate, ethyl butyrate, ethyl caproate, ethyl caprylate, and ethyl lactate), alcohols (isobutanol, butanol, isoamyl alcohol, phenylethyl alcohol, and furfuryl alcohol), carboxylic acids (acetic, propionic, butyric, and caproic acids), ketones (3-hydroxy-2-butanone, furfural, benzaldehyde, and phenylacetaldehyde), and phenols (guaiacol and tetra-ethyl guaiacol) (Niu et al., 2017). These substances usually have special aromas, such as fruity short-chain fatty acid esters; floral aromatic compounds; nutty pyrazines; cellar bottom: medium- and short chain fatty acids (Li et al., 2017). Baijiu flavor substances are produced by fermentation microbiota via enzyme degradation and microbial interactions. Understanding the mechanisms of these processes is important to unravel how Baijiu flavor profiles develop.

**Table 2:** Effects of microbiota on the formation of flavor compounds in Jiang-flavored baijiu.

Microbiota	Species	Flavors	References
Bacteria	*Bacillus subtilis* LBM 10019 and *Bacillus vallismortis* LBM 10020	L-cysteine, 2-furfuryl mercaptan	Shen et al. (2020)
Bacteria	*Bacillus amylus* XJB-104	Tetramethylpyrazine	Zhang et al. (2019)
Bacteria	*Bacillus subtilis*	2,3-butanediol, 4-methylpyrazine and acetylmethyl methanol	Jin et al. (2017), Sun et al. (2016)
Bacteria	*Acetobacter*	Ethyl acetate	Wu et al. (2012a)
Bacteria	*Bacillus cereu*	Ethyl hexanoate	Zhao et al. (2017)
Bacteria	*Lactobacillus*	Lactic acid and ethyl lactate	Liu et al. (2014)
Bacteria	*Gluconobacter* and *Acinetobacter*	Acetic acid	Wang L. et al. (2021)
Bacteria	*Enterococcus, Clostridiales and Acinetobacter*	(2E)-2,3-dimethyl-2-pentenoic acid, methyl hexadecanoate, methyl oleate, methyl tetradecanoate, methyl linoleate and ethyl hexadecanoate	Jin et al. (2019)
Yeast	*Zygosaccharomyces*	Ethylhexanol, decanoic acid, dodecanol, 2-nonanol, lauric acid, octanoic acid, acetophenone, ethyl octanoate, and 4-tert-butylphenol	Zhuang et al. (2017)
Yeast	*Saccharomyces cerevisiae*	Ethanol, 2-phenylethanol, and *β*-glucosidase	Zha et al. (2018)
Yeast	*Paecilomyces variotii*	Phenylethanol, phenylacetic acid, ethyl hexadecanoate, ethyl linoleic acid, ethyl oleate	Wang (2022)
Fungi	*Aspergillus, Monascus*, *Rhizopus*, and	Ethyl hexanoate	Li et al. (2019)
Fungi	*Mucor* sp.	Ethyl linoleate and ethyl oleate	Han et al. (2012)
Fungi	*Paecilomyces*	Alcohols, aldehydes, acids, and esters	Huang et al. (2014)

Enzymatic degradation and production of Baijiu flavor substances are mediated by secreted enzymes, including lipase, amylase (*α*-amylase, β-amylase, saccharifying amylase and *γ*-amylase), cellulase, esterification enzymes, pectinase, and tannase. During solid-state fermentation (Li et al., 2019). These enzymes accelerate the conversion of starch, cellulose, protein and fat to smaller molecular weight flavor substances such as glucose, fructose, fatty acids, and esters (Zhu et al., 2022). In sorghum, α-amylase in amylase reacts amylopectin to produce dextrin, maltose, and glucose, and with amylose to produce oligosaccharides, maltose and glucose (Li et al., 2019). Cellulase (produced by *Bacillus subtilis*, *Trichoderma*, *Aspergillus* and *Penicillium*) hydrolyzes lignin and cellulose; protease hydrolyzes protein into amino acids, the precursor of other Baijiu flavor substances; and the amino acids also provide nutrients to support the growth of microbes in the community. Lipase decomposes fat in raw materials into glycerol, fatty acids, monoglycerides and diglycerides (Li et al., 2019).

In addition to enzymatic degradation, complex microbial interactions also contribute to the formation of flavor substances in Baijiu fermentation (Meng et al., 2015). Mutualism, parasitism, antagonism, predation, and competition among Baijiu-making microbiota inhibit some populations and induce others, influencing the population structure in different Baijiu manufactories and generating different flavor profiles. For instance, populations that include *Aspergillus oryzae XJ10* and *Saccharomyces cerevisiae* produce more esters, aldehydes, ketones, and ethyl acetate than either species alone, indicating that *Aspergillus oryzae XJ10* and *Saccharomyces cerevisiae* produce a synergistic effect and unique fermentation product (Chen et al., 2014). Other microbes, in the Baijiu fermentation system can interfere with the growth of some fermenting populations. For instance, *Saccharomyces cerevisiae CCTCCM2014463* inhibits the growth of *Zygosaccharomyces bailii CGMCC4745*, but has no significant effect on *Bacillus licheniformis CGMCC3962* and *Lactobacillus buchneri* (Xu et al., 2018). Similarly, *Saccharomyces cerevisiae* is also able to impact the growth of *Lactobacillus bread* when co-existence with each other, the presence of *Saccharomyces cerevisiae* in fermentation system is available to produces ethanol and inhibits the growth and reproduction of *Lactobacillus bread* and lactic acid accumulation (Liu et al., 2022a).

## 4 Input of Jiang-flavor-baijiu fermentation microbes from the surrounding environmental matrices

Regarding the source of Jiang-flavor-Baijiu fermentation microbes, many efforts demonstrated that Baijiu fermentation microbe residue in the fermented grain samples, high-temperature Daqu, and sealed mud pits are readily input from the production workshop of Jiang-flavor-Baijiu (Hao et al., 2021; Wang M. Y. et al., 2018). For instance, it was reported that the predominant fungi (*Monascus ruber* and *Hanseniaspora* var*oom*) responsible for Jiang-flavor-Baijiu fermentation, the predominant bacteria (e.g., *Weissella cibaria*, *Weissella cibaria*, and *Microbacterium testaceum*) were originated from atmospheric, water, or soil of Daqu, outside the Baijiu manufacturing workshop (Wang et al., 2016). Tracking the source of Chinese Jiang-flavor-Baijiu fermentation microbes is important elucidating their unique flavor as microbial metabolism. Herein, we systematically overviewed the sources of Jiang-flavor-Baijiu fermentation microbiota from surrounding environmental matrices.

### 4.1 Soil transportation of baijiu fermentation microorganisms

The unique soil types of Jiang-flavor-Baijiu production workshops include red soil (purplish red mudstone, purplish red rock and soil and mixed purplish red clay) and yellowish brown soil (yellowish brown sandstone and clay) (Li et al., 2017). These soils contain abundant minerals and trace elements that support microbial growth and reproduction. A recent study showed that the content of rare-earth elements (REE) (i.e., Samarium (Sm), Europium (Eu), Erbia (Er), and Cerium (Ce)) in the surface soil Jiang-flavor-Baijiu producing area (287.1 μg/g) was than that in soil (186.8 μg/g) and crust (207 μg/g) for other areas of mainland China (Jiang et al., 2023). The rich mineral elements in the soil can improve the quality of Baijiu: by increasing the mineral content of the water, composing sealed mud pits and cultivating high-quality raw materials. Soil minerals support Baijiu fermentation microbiota and improve the quality of water, sealed mud pits, and raw materials, thus indirectly improving Baijiu quality.

The sealed mud pits are considered as the ideal habitat of fermentation microbiota. Under sealed fermentation conditions, different microbiota in the sealed mud pits continue to metabolize and affect Baijiu flavor (Li et al., 2023). During Jiang-flavor-Baijiu fermentation, the purple red mud was extensively utilized as sealed pit mud due to its specific signatures, including low sand content, strong viscosity, and well sealing performance. In addition, the purple red mud is of moderate in acid and alkali, rich in a variety of beneficial components (organic matter content ~1%), and rich in mineral nutrients (SiO_2_: 52–74.7% (average 60.1%); Fe_2_O_3_: 5.28–7.43% (average 6.51%); Al_2_O_3_: 11.7–17.6% (average 15.6%)) (Liu and Tan, 2011). This will facilitate for Baijiu fermentation microbial growth and community succession, indicating that soil quality is an important determinant of high-quality Jiang-flavor-Baijiu fermentation. A recent study investigated the microbial community structure in fresh red soil, sealed mud pits used for 1 month and sealed mud pits used for 12 months near the producing area of Jiang-flavor-Baijiu, and discovered abundant microbiota residue (Wang et al., 2015a). The abundance of microbial populations was greater in fresh soil used for sealed mud pits (81 families) than in sealed mud pits used for 1 month (77 families) and 12 months (47 families), suggesting the number of microbial species decreased with increasing age of Maotai red soil as sealed mud pits (Wang et al., 2015a). In addition, there are 12 families of microbiota were detected in fresh soil and in sealed mud pits used for 1 and 12 months (Wang et al., 2015a). During the long-term domestication of microbiota in cellars, the content of high-efficiency fermentation microbiota continues to increase, an important determinant of improved Baijiu quality. Hence, in red and yellow soil surrounding the Jiang-flavor-Baijiu manufacturing workshop can be used as sealed mud pits and transfer abundant Baijiu fermentation process, influencing the Baijiu flavor over the long-term.

High-quality red and yellow soil surrounding the Baijiu manufacturing workshop yields high-quality raw materials essential to the Baijiu fermentation microbial community and flavor because these materials support the energy and habitat for microbial growth. The diverse content of amylopectin and amylose in sorghum from different Baijiu-producing areas generates differences in water absorption and gelatinization capacities during the manufacture of Baijiu and koji, affecting saccharification efficiency and other differences in the community structure of Baijiu-making microbiota. In addition, differences in starch content and structure in different types of sorghum will also lead to a variety in the composition and structure of amino acids after enzymatic hydrolysis, thereby affecting the content and composition of flavor substances (alcohols, aldehydes, carboxylic acids and esters) (Ding et al., 2024; Ma et al., 2022). Hongyingzi sorghum used in Jiang-flavor-Baijiu brewing is a unique, organic waxy sorghum characterized by its small grain, thick skin and full grain. The total starch content of Hongyingzi sorghum is >65%, and amylopectin accounted for 88–93% of the total starch content, giving it characteristics of small grain, thick skin, full and solid, and able to withstand nine rounds of cooking. In contrast, the Hongyingzi sorghum in which mainly produced in Renhuai, Guizhou, China (105°–106 °E and 27°–28° N latitude) are planted with mid-subtropical monsoon climate conditions (average annual temperature of 18°C). This further induced the Hongyingzi sorghum became a symbol as the raw material of the unique flavor Jiang-flavor-Baijiu.

### 4.2 Aquatic transport of baijiu fermentation microorganisms

The water for Jiang-flavor-Baijiu fermentation comes from the Chishui River Basin, which has an intact natural ecology and exiguous industrial and anthropogenic activity. In addition, the Chishui River contains many trace elements beneficial to human health that are also discovered in red soil, leading to the high-quality Jiang-flavor brew water being moderate in acidity and rich in minerals and trace elements (Li et al., 2017). The basic Chishui River water quality parameters are as follows: pH: 6.5–8.5, total hardness: 2–7 mmol/L, nitrate nitrogen: <0.2 mg/L, free chlorine residual: < 0.1 mg/L, consistent with high-quality brew water requirements (pH: 8.1, total hardness: 6.2 mmol/L, nitrate nitrogen: 0.5 mg/L, free chlorine residual: < 0.3 mg/L) (Li et al., 2017). Previous effort has focused on the microbial community structure in the Chishui River, and discovered substantial fermentation microbiota residue (Jin et al., 2009), thereby unravel the preliminary fermentation microbiota in environmental media is necessary to improve the quality of Baijiu. Jiang-flavor-Baijiu fermentation microorganisms *Actinobacteria*, *Bacteroidetes*, *Proteobacteria*, and *Firmicutes* have been detected year-round in the Chishui River using 16S rRNA gene sequencing analysis. *Proteobacteria* was the dominant species, followed by *Firmicutes*, *Actinobacteria*, and *Bacteroidetes* (Tan et al., 2014). The microbial community structure in the Chishui River and water intake sites at Baijiu manufactories also revealed the presence of Baijiu fermentation microbiota, including 55 phyla, 167 classes, 415 orders, 706 families, and 1,431 genera. *Pseudomonas* (16.99%) and *Massilia* (12.45%) were the dominant species (Lv et al., 2021).

However, during long-term Baijiu manufacturing activities, the discharge of wastewater will influence nearby water quality despite wastewater treatment efforts, affecting water quality (pH), chemical oxygen demand (COD), and ammonia nitrogen (NH_3_-N) and possibly influencing the brewing microbial community structure. Using COD and NH_3_-N as example, a recent study discovered that the quality of the water intake at Maotai Distillery is significantly different from that of the upstream/downstream water intake, with COD concentration 7.22 ± 0.01 mg/L in the water body at the intake section of the distillery less than the upstream 8.62 ± 0.07 mg/L and downstream concentrations 8.36 ± 0.05 mg/L. The NH^3^-N concentration 0.51 ± 0.02 mg/L in the Baijiu water intake is greater than upstream 0.28 ± 0.01 mg/L and downstream concentrations 0.33 ± 0.01 mg/L (Lv et al., 2021). This is due to the fact that continuous Jiang-flavor-Baijiu making activities inevitable discharge varied organic contaminants (fertilizers and natural manure) into the water, accelerating oxygen the consumption and the formation of NH^3^-N, leading to the COD and NH^3^-N in the Baijiu making water intake section higher than that of the upstream river. Simultaneously, the COD and NH^3^-N associated contaminants produced by Baijiu making areas are readily to be degraded and diluted during the surface runoff, resulting the COD and NH^3^-N concentration Baijiu making water intake section higher than that of the downstream river consequently (Lv et al., 2021). Redundancy analysis showed that the concentration of COD_Mn_, COD, and DO in the water of Jiang-flavor-Baijiu fermentation areas also determined the diversity of microbial community structure. Taking DO as an example, *unclassified_Comamonadaceae*, *Sphingorhabdus*, *Flavobacterium*, and *Pseudarcicella* were significantly positively correlated with DO (*p* < 0.05), while *unclassified_Micrococcaceae* and *Pedobacter* were significantly negatively correlated with DO (*p* < 0.05), verifying that water quality is an important determinant of the structure and function of bacterial communities in the ecosystem surrounding Baijiu brewing areas (Lv et al., 2021).

There were significant differences in the microbial community structure in the water body of the Baijiu brewing area and upstream/downstream water of the water intake, with *Pseudomonas* (15.33%) and *Acinetobacter* (14.99%) being the dominant species. *Acinetobacter* (22.21%) and *Flavobacterium* (16.84%) were the dominant microbiota upstream of the water intake, while *Pseudomonas* and *Massili* were the dominant microbiota downstream of the water intake (Lv et al., 2021). We conclude that long-term Baijiu-making activities have led to a significant difference in water quality between Baijiu regions and waters upstream and downstream of the manufactory intakes. This difference drives the specific distribution of microbial community structure in Baijiu regions. The intake water is rich in minerals that have an important impact on Baijiu flavor. The content of sodium (21,000 μg/L), magnesium (67,000 μg/L), chlorine (27,000 μg/L), manganese (14.47 μg/L), and iron (164.4 μg/L) in the water of Jiang-flavor Baijiu is much higher than that of Luzhou Laojiao (sodium: 12783 μg/L, magnesium 5,886 μg/L, chlorine 22,776 μg/L, manganese 3.77 μg/L, and iron 37.9 μg/L) and Wenjun Distillery (sodium 8,065 μg/L, magnesium 5,103 μg/L, chlorine 10,773 μg/L, manganese 2.1 μg/L, and iron 23.9 μg/L), indicating that there are abundant mineral elements in the water of Jiang-flavor-Baijiu (Wang et al., 2022a). These inorganic salt ions improve the formation of flavor substances and improve the quality of Baijiu during fermentation.

### 4.3 Atmospheric transport of baijiu fermentation microorganisms

As with water, microbiota in the air are also an important source of microbiota in Jiang-flavor-Baijiu. A recent study explored the dominant microorganism species in the air around the Jiang-flavor Baijiu brewing area and found a relativity stable microbial community structure that varied with the seasons, such as *Acinetobacter*, *Arthrobacter*, *Aspergillus*, *Bacillus*, *Brevibacterium*, *Cedecea*, *Pichia*, and *Rhodoceccus* (Wang et al., 2022a). This variability may be due to the unique geographical location of the Jiang-flavor-Baijiu brewing area, which is surrounding by mountains. The airflow is slow (wind speed 1.3–1.35 m/s), the annual average temperature fluctuates little (the average temperature from 1961 to 2018 was 15.1°C, SD (standard deviation) 0.34°C), and the annual average precipitation fluctuates stably (average precipitation from 1961 to 2018 was 1018.6 mm, SD 10 mm), resulting in a stable microbial community structure in the area and reduced influence of external environment (Wang et al., 2022a). The primary bacteria in the air of Jiang-flavor-Baijiu brewing areas are *Streptomyces*, *Acinetobacter*, *Staphylococcus*, *Bacillus*, *Brevibacterium*, *Brevibacterium*, *Kocuria*, and *Pseudomonas*. The dominant fungi are *Aspergillus*, *Cladosporium*, and *Pteris*. Thus, fermentation microbiota residue in the Jiang-flavor Baijiu brewing area originated from surrounding environment, highlighting the critical impact of brewing microbial community structure on the flavor of Jiang-flavor Baijiu. For example, For example, Zhou et al. (2024) discovered that bacterial communities of phyla *Actinobacteriota*, *Firmicutes*, and *Proteobacteri*, as well as fungal communities of *Ascomycota* and *Basidiomycota* were predominant airborne microbiomes residue in the Jiang-flavor Baijiu core production areas, whereas the *Saccharomonospora*, *Thermoactinomyces*, *Bacillus*, *Oceanobacillus*, and *Methylobacterium* contributing were demonstrated as core functional flora to the Baijiu producing according to the Random Forest analysis (Zhou et al., 2024). Relative *Bacillus* amounts in the air surrounding the Baijiu-making workshop, koji-making workshop, and outdoor air surrounding the workshop were also assessed in the Daqu, sealed pit mud, and fermented grains (accumulated fermented grains and pit fermented grains), including *B. licheniformis*, *B. amyloliquefaciens*, *B. subtilis, Paenibacillus lactis*, *Bacillus lentus, Paenibacillus* sp., and *Staphylococcus lentus.* Fermentation microbiota in the Daqu, pit mud and fermented grains exhibited obvious homology with the microbial community structure of surrounding air, indicating that air exposure is an important contributor to Baijiu-fermentation microbiota.

## 5 Environmental driving factors controlling the Jiang-flavor baijiu fermentation microbial diversity

### 5.1 Effect of natural geographical and meteorological conditions on the fermentation microbial community structure

To bridge the knowledge gap unraveling the role of environmental factors (monsoon climate, temperature, wind speed, humidity, and precipitation) on shaping microbiomes during Jiang-flavor-Baijiu brewing, correlations between environmental factors and microbial community structure indicators (ACE index and Chao index) were thus established (Wang et al., 2022b), which favoring to understood the bacterial diversity and community dynamics during Jiang-flavor-Baijiu open fermentation process. For instance, Beecroft et al. (2012) developed the microbial communities surrounding the Jiang-flavor manufacturing workshops (indoor dust, windows, sills, and wall surfaces) using high throughput sequencing analysis (Beecroft et al., 2012). They discovered 33 bacterial classes (*Gammaproteobacteria*, *Bacilli*, *Sphingobacteriia*, *Alphaproteobacteria*, *Actinobacteria*, *Flavobacteriia*, and *Betaproteobacteria*), 153 bacterial families (*Enterobacteriaceae*, *Sphingobacteriaceae*, *Bacillaceae*, *Flavobacteriaceae*, *Thermoactinomycetacea*e, *Planococcaceae*), and 396 bacterial genera (*Sphingobacterium, Enterobacter*, *Pantoea*, *Acinetobacter*, *Oceanobacillus*, and *Pseudomonas*) (Beecroft et al., 2012). The bacterial community structure was significantly correlated with the surrounding environment (temperature, sunlight, humidity, atmospheric pressure, and precipitation) (Wang et al., 2022b). The unique natural environmental factors and their influence on Baijiu fermentation microbial communities were systematically reviewed.

Temperature is important in microbial dynamics and diversity during fermentation, especially in the Daqu making (Garcia et al., 2023; Simon et al., 2020; Walker et al., 2018; Zhang et al., 2023). *Alphaproteobacteria*, *Acetobaqcteraceae, Sphingomonadaceae*, *Caulobacteraceae*, and *Brevundimonas* were positively correlated with the fermentation temperature, with *p* values were 0.350, 0.488, 0.283, 0.290, and 0.284, respectively. In contrast, *Bacillaceae* (*p* = 0.489), *Bacillus* (*p* = 0.387), *Oceanobacillus* (*p* = 0.419), *Thermoactinomycetaceae* (*p* = 0.284), and *Kroppenstedtia* (*p* = 0.347) exhibited a negative correlation with fermentation temperature (significance defined as *p* < 0.5) (Beecroft et al., 2012). These results highlighted the important role of temperature in the domestication and screening of microbial community structures during fermentation. Jiang-flavor Baijiu manufacturing facilities are located in the Guizhou province, southwest China, which is a subtropical climate with high temperature and humidity that facilitate microbial growth. The temperature of Jiang-flavor-Baijiu manufacturing workshop is reported as in the range of 2.7°C–40.6°C, with an annual average temperature of 17.4°C, which provides a suitable habitat for the growth and reproduction of fermentation microbiota (Li et al., 2017).

A seasonal subtropical climate brings precipitation (annual precipitation from 1961–2018: 1018.6 ± 10 mm), resulting in a relatively high air humidity to the Jiang-flavor Baijiu making regions, and influencing the Baijiu brew microbial growth and propagation (Li et al., 2017). The abundance of *Gammaproteobacteria* (*p* = 0.342), *Enterobacteriaceae* (*p* = 0.376), *Escherichia-Shigella* (*p* = 0.366), and *Pantoea* (*p* = 0.367) were positively correlated with humidity, while *Rhodospirillales* (*p* = 0.363), *Acetobacteraceae* (*p* = 0.298), and *Actinobacteria* (*p* = 0.359) were negatively correlated with humidity (Wang et al., 2022b). *Bacillaceae*, *Lentibacillus*, *Thermoactinomycetaceae*, and *Lactobacillaceae* were also reported to correlate significantly with precipitation. Thus, humidity and regional precipitation significantly affect Baijiu fermentation microbial diversity. Microbial respiration and growth decrease with decreasing air humidity and temperature (Cruz-Paredes et al., 2021).

Moreover, the geology of Jiang-flavor-Baijiu regions creates unique wind speed and direction conditions that drive seasonal variations in the type and abundance of Baijiu brew microbial species and Baijiu quality (Tan et al., 2014). Microbes in air influence Baijiu manufacturing and a steady wind direction and speed facilitate consistent transfer of microbes from the air to the fermentation raw material, especially during open static fermentation of Jiang-flavor Baijiu. Wang et al. (2022b) characterized the variations of wind speed in a Jiang-flavor manufacturing workshop (Chishui River basin, China) from 1961- to 2018, and discovered that the multi-year wind speed variation is 1.33 m/s, with the wind speed increasing at 0.014 m s^−1^ every 10 years (Wang et al., 2022a). The Jiang-flavor-Baijiu manufacturing workshop is located in a closed basin area (103°36′–109°35’N, 24°37′–29°13′E) surrounding by mountains, and has low wind velocities. This unique topography favors stability in the Baijiu brew microbial community structure and facilitates stable flavor and quality in the product (Jiang et al., 2009). The Jiang-flavor-Baijiu manufacturing workshops are located in low-latitude mountainous areas and are subject to subtropical monsoons. The climate is warm and humid, with sufficient rainfall to support the growth of fermentation microbiota. In addition, the Jiang-flavor-Baijiu factories are located in a basin that is relatively closed, supporting long-term stability in the wine-making microbial community structure and essential to the high quality of Jiang-flavor-Baijiu. Thereby, from the perspective of environmental microorganisms, we can conclude that long-term Baijiu making activities and regional suitable climatic conditions will lead to a relatively stable microbial community structure in the water, air and soil surrounding the Baijiu producing area, resulting the quality of Baijiu produced by new distilleries is not as good as that produced by old distilleries. This is assigned to the close correlation among the regional geological structure (location, wind, humidity, and water quality), Baijiu brewing microbial community structure, composition of microbial metabolites, and liquor flavor. These also highlighted the fact protecting the ecology of Jiang-flavor-Baijiu plays essential role in maintaining the stability of Baijiu fermentation microbial community and their special flavors.

### 5.2 Environmental factors controlling microbial succession within fermentation system

Environmental factors driving the microbial community structure are also varied within the Baijiu fermentation system. For instance, Yang et al. (2023) developed the changes of microbial community structure succession during 1–6 rounds of Jiang-flavor-Baijiu stacking process and discovered that, with the continuous progress of the stacking process, the dominant bacterial genera are readily to be replaced from *Lactobacillus* to *Acetobacter*, while the fungal genus are readily to be replaced from *Pichia* to *Candida* (Yang et al., 2023). With the Jiang-flavor-Baijiu fermentation transfer from the stacking heap fermentation to pit fermentation, microorganisms also changed consequently according to the high-throughput sequencing analysis. The abundance of *Pichia* decreased whereas the abundance of Saccharomyces began to increase and act as one of the dominant fungal species within the fermentation system. Meanwhile, in comparison with stacking heap fermentation, the bacterial species of *Lactobacillus* and *Saccharomyces* increased continuously, and *Lactobacillus* contributing >80% to total bacterial abundance during pit fermentation process (Hao et al., 2021). Environmental driving factors controlling the successive changes of microbial communities during Jiang-flavor-Baijiu brewing are responsible to the multiple constituents of temperature, moisture, sugar content, ethanol, acidity, lactic acid (Yang et al., 2023). Specifically, lactic acid, ethanol, acidity, and temperature were found to positively correlated with *Lactobacillus* and *Saccharomyces;* acetic acid was demonstrated to negatively controlled the abundance of *Virgibacillus* and *Bacillus*; and water was reported to negatively controlled the abundance of *Virgibacillus, Bacillus, Oceanobacillus, taphylococcus,* and *Kocuria* simultaneously (Hao et al., 2021). These highlighted a comprehensive environmental factors for natural meteorological and Baijiu fermentation system on the specific Baijiu brewing microbial succession.

## 6 Conclusions and perspective

Jiang-flavor-Baijiu is one of the most complex and typical Chinese liquor in which produced through traditional open brewing. Nevertheless, the source of the Jiang-flavor-Baijiu fermentation microbial community current still unclear, hindering the subsequent knowledge for the procedure of microbial metabolism and the formation of specific flavor substances in Baijiu liquor. This study systematically overviewed the microbial sources in various stages of Maotai flavor Baijiu. The obtained results revealed that microbes residue in the Jiang-flavor-Baijiu fermentation system are prone to be originated from the surrounding environmental matrices, with the relationships between Baijiu fermented microbial diversity and surrounding meteorological and geographical conditions (temperature, humidity, solar irradiation, precipitation) has been widely demonstrated according to the current knowledge. Specifically, environmental microorganisms in the air, water, and soil of Baijiu producing area are readily enter into the Baijiu manufacturing plants via air flow, sealed mud pits, or water intake. This will further interfere the microbial community structure in such as raw materials, Daqu-making, stacking fermentation, distillation, and pit fermentation in sequential, and ultimately shaping the special flavor of liquor. These results offer a comprehensive understanding elaborating the source of microbes among various stages of Maotai flavor Baijiu brewing. In contrast, the obtained results also highlighted that: protecting the regional ecological environment quality of Jiang-flavor-Baijiu producing area is helpful to maintain the stability of the Baijiu fermentation microbial community structure during the brewing process, and maintains the quality and unique flavor of the liquor.

Further research should explore the correlations and interactions between environmental conditions and Jiang-flavor-Baijiu fermentation microorganism communities. Regular monitoring of water, air, and soil qualities surrounding the Jiang-flavor-Baijiu making area will support efforts to maintain Baijiu quality and flavor. It is also important to establish a system of environmental protection measures to maintain the stability of the fermentation microbial community and eliminate potential point source and non-point source pollution in brewing areas. Finally, it is necessary to explore the spatiotemporal diversity of microbial community dynamics and environment factors in Jiang-flavor Baijiu fermentation.
